# Rate of Missed Intracranial Colloid Cysts on MRI and CT

**DOI:** 10.7759/cureus.49004

**Published:** 2023-11-18

**Authors:** Ayat A Sharif, Erik DiGiacomo, Marcin Czarniecki, Anousheh Sayah

**Affiliations:** 1 Neuroradiology, Georgetown University School of Medicine, Washington, USA; 2 Neuroradiology, MedStar Georgetown University Hospital, Washington, USA

**Keywords:** ct and mri brain, detection, hydrocephalus, foramen of monroe, colloid cyst

## Abstract

Background and purpose: Intracranial colloid cysts of the third ventricle are rare; however, they may be symptomatic. They can create a mass effect on the foramina of Monro, resulting in acute hydrocephalus. Colloid cysts are detectable on CT and MRI but are commonly missed. In this paper, we investigate the rate of missed colloid cysts on MRI and/or CT imaging within our multihospital metropolitan medical group.

Materials and methods: A retrospective, institutional review board-approved search of the network-wide picture archiving and communication system (PACS) from January 1, 2010, to October 31, 2020, was performed to identify reports including a "colloid cyst" in MRI brain or CT head imaging. Results without imaging and/or surgical confirmation of intracranial colloid cysts were excluded, rendering 229 cases. A PACS review of these cases was performed by two neuroradiologists to determine instances where the cyst had previously been imaged but not diagnosed on either CT or MRI.

Results: Two hundred twenty-nine subjects had confirmed colloid cysts through imaging and/or surgical reports. Of these, 46 had prior imaging depicting a colloid cyst either on CT and/or MRI without mention on the interpretative report, resulting in a non-detection rate of 20.1%.

Conclusion: Intracranial colloid cysts can be missed at a considerably high rate, which is concerning given their clinically unpredictable nature and ability to cause significant morbidity and mortality. As such, it is important to take a proactive approach to searching for these cysts as part of a regular imaging search pattern and to continue to determine new methods of increasing detection sensitivity.

## Introduction

Colloid cysts are benign intracranial primary ventricular cystic neoplasms. There is disagreement regarding the origin of colloid cysts. According to Czervionk et al., colloid cysts are derived from neuroepithelium [1], while Ho and Garcia provide evidence for an endodermal origin of colloid cysts [2]. Colloid cysts have a prevalence rate of 0.1% to 1% of all intracranial tumors [3]. Grossly, colloid cysts have a gelatinous texture. They often arise in the anterior or anterosuperior segment of the third ventricle, anterior to the foramina of Monro [4,5]. This location gives colloid cysts the potential to cause obstruction, preventing the flow of CSF to the cerebral aqueduct [4]. Only four cases have been documented of colloid cysts arising in the fourth ventricle [6].

The clinical presentation of a colloid cyst is heterogeneous and can vary widely from patient to patient. While most patients remain asymptomatic, others can experience significant symptoms such as diplopia, gait disturbance, vertigo, nausea and vomiting, and paroxysmal headaches [7], to name a few. Their location adjacent to the foramina of Monro can, on occasion, result in sudden obstructive hydrocephalus and can present with a thunderclap headache or unconscious collapse. The headaches tend to be positional, and patients may learn how to relieve their symptoms. Due to the obstructive nature of colloid cysts, they have also been implicated as a cause of sudden death. There has also been documentation of colloid cysts with hemorrhagic properties [8], underscoring the importance of early diagnosis. Treatment of a colloid cyst is a medical necessity in preventing obstructive hydrocephalus, ventriculomegaly, and subsequent mortality [9]. Therefore, colloid cysts are a preventable cause of mortality in patients of all ages.

The management of colloid cysts varies with surgeon preference and radiologic findings. A microsurgical approach that is transcallosal and transforaminal carries the lowest risk of iatrogenic damage to surrounding structures, including the fornix and the thalamus. If there is obstruction and subsequent dilation of the ventricles, endoscopic management is preferred [5]. However, there is a strong indication for surgical intervention in cases of colloid cysts that present with symptomatology [10].

The definitive diagnosis of a colloid cyst requires imaging. Modalities that have historically successfully imaged colloid cysts are CT and MRI. CT and MRI are both imaging modalities that have proven useful in determining the nature of colloid cysts. The composition of colloid cysts can vary; some are rich in cholesterol or protein, while others have calcifications or hemorrhage [11]. For that reason, there is some variability in the density of colloid cysts on CT and the intensity of colloid cysts on MRI, with variations in visualization on different sequences.

On CT imaging, most colloid cysts appear hyperdense, but there are instances in which they appear isodense and rarely even hypodense. Ventriculomegaly may also be observed in cases of obstruction [9]. On MRI, different colloid cysts will have varying intensities on visualization, and there is further variation within the different MRI sequences. Cysts that are rich in protein and cholesterol are hyperintense on T1-sequences and hypointense on T2-sequences. As cholesterol content increases in colloid cysts, the appearance on MRI would show greater hyperintensity on T1-sequences and a decrease in hypointensity on T2-sequences. Colloid cysts that are hypointense on T2-sequences cannot be visualized on fluid-attenuated inversion recovery (FLAIR). These cysts are difficult to aspirate due to an increase in viscosity [11]. There have also been pilot studies that evaluated the "black rim susceptibility" sign on susceptibility weighted imaging (SWI) as a novel finding for the accurate diagnosis of colloid cysts [12].

The characteristics of colloid cysts make them difficult to identify and detect by radiologists. Colloid cysts may be small, are most often located medially, and have variable imaging attributes due to the variability in composition. For that reason, we evaluated the incidence of colloid cysts being missed on interpretation reports for different imaging modalities, including CT and MRI, in our multi-hospital metropolitan medical group through a retrospective chart review.

## Materials and methods

The Institutional Review Board of Georgetown University Medical Center, Washington, D.C., USA, granted expedited approval of this study (approval number: STUDY00003891, approval date: 6/24/2021) to allow for the search and review of patient records via the multi-hospital radiology picture archiving and communication system (PACS) network. This study was performed in line with the principles of the Declaration of Helsinki. Our PACS was queried for the phrase "colloid cyst" in instances of CT head or MRI brain during the period from January 1, 2010, to October 31, 2020. This initial search generated 603 studies, which were then merged into 298 unique patients by our search engine. Of these, 69 patients were excluded because they did not have an imaging or surgical history of an intracranial colloid cyst.

The PACS imaging charts for the remaining 229 patients were reviewed for basic demographic data, including gender and age, as well as all previous CT and MRI studies of the brain. Charts were reviewed by two board-certified neuroradiologists with 10 years of experience and a second-year neuroradiology fellow concurrently in unison and evaluated for prior imaging studies that had depicted the colloid cyst but were not reported on the associated interpretative report. From these studies, we collected the following data: greatest anterior-posterior (AP) x transverse size of the lesion on axial images (in mm); relative density of the lesion if missed on CT to surrounding brain parenchyma (hypo/iso/hyperdense); relative intensity of the lesion if missed on MRI to surrounding brain parenchyma on T1 and T2 (hypo/iso/hyperintense); shape of lesion (round/ovoid or irregular); presence of hydrocephalus on the images; fellowship training of the interpretative radiologist (neuro or non-neuro); clinical indication for study; and the presence of hydrocephalus at the time of missed imaging. Of note, patients with multiple prior studies in which their lesion was missed were counted as a single instance of a missed cyst, as these multiple misses can likely be attributed to the inherent characteristics of the colloid cyst on imaging.

The data was tabulated into a database, and then statistical analysis followed. Data analysis included percentage calculation of the colloid cyst miss rate through a ratio of studies of missed colloid cysts and the total studies that were read; average calculation of the dimensions of missed cysts; mean/median/quartile age calculations of all patients with colloid cysts; mean time to detection of cysts after initial miss; percentage of neuroradiology fellowship training in radiologists who missed cases; clinical indication for each imaging study that missed the diagnosis of colloid cyst (headache, trauma, altered mental status (AMS), stroke, assess ventriculaperitoneal (VP) shunt, syncope, vertigo, and unknown); and presence of hydrocephalus at the time of study.

## Results

A total of 229 subjects with confirmed imaging cases of intracranial colloid cysts were evaluated, and their previous imaging was reviewed in our multi-hospital PACS system in the 10-year period from January 2010 to October 2020. The mean age of this group at the initial diagnosis was 52.3 years ± 19 years, with a range of 12 to 96 years. The median was 52 years, with an interquartile range of 25th and 75th percentiles being 39 years to 65 years. There were 133 males and 96 females (58% male). On retrospective review, 46 of our 229 patients (20.1%) had imaging evidence of an intracranial colloid cyst on prior imaging that had not been reported on either CT or MRI (Table 1). Of the missed studies, two were missed on MRI only, 40 were missed on CT only, and four cases were missed on both CT and MRI (Table 1). The mean time from which colloid cysts were initially imaged (and retrospectively detected) to their ultimate detection on imaging was 3.25 years. The missed cases were interpreted by a mix of non-neuroradiology fellowship-trained radiologists as well as neuroradiologists, with 53% of missed cases read by non-fellowship-trained imagers.

**Table 1:** Incidence of missed colloid cysts on imaging by unique subject

Total number of unique subjects with colloid cysts reviewed	229 (subject)
Male	133
Female	96
Missed cases	46
Missed on CT only	40
Missed on MRI only	2
Missed on CT and MRI	4
Rate of cysts missed by patient	20.1%

Characteristics of missed colloid cysts included small size, with the average cyst measuring 5mm AP x 5mm transverse (SD ± 1.5 x 1.6mm) on axial imaging (range: 2-10mm AP x 2-9mm transverse) (Table 2), and irregular shape, with 19 of the missed colloid cysts on CT demonstrating non-round/ovoid margins (Table 3). Of the cases missed on CT, 43/44 (98%) were hyperdense to brain parenchyma, with the one remaining being isodense (Table 3). Two cysts were missed on MRI, and in both cases, the cysts were hyperintense on T1- and T2-weighted imaging.

**Table 2:** Size dimensions of missed colloid cysts AP: anterior-posterior

AP diameter (mm)	
Average	4.6 ± 1.5
Minimum	2
Maximum	10
Transverse diameter (mm)	
Average	4.9 ± 1.6
Minimum	2
Maximum	9

**Table 3:** Imaging features of missed colloid cysts on CT only

Missed on CT	44 (case)
Hyperdense	43
Isodense	1
Hypodense	0
Round/ovoid	25
Irregular	19

The clinical indication for each study that had missed colloid study identification on the imaging report was stratified into one of seven categories, which are stroke, altered mental status, trauma including falls, syncope, vertigo, assess VP shunt, and headache (Table 4). For those studies in which headache was the indication, the presence of hydrocephalus was tabulated, as was whether the subject had a VP shunt already in place. In one study in which the diagnosis of colloid cyst was missed on imaging for a subject with a headache, the subject did end up undergoing colloid cyst resection subsequently after one year of detection and repeat imaging. Three of the nine headache cases that had missed the diagnosis of the colloid cyst presented features of hydrocephalus. One of the nine cases that had missed a colloid cyst read had a VP shunt in place with features of hydrocephalus.

**Table 4:** Clinical indication for imaging of subjects that had a missed colloid cyst on report VP: ventriculoperitoneal, AMS: altered mental status

Total missed colloid incidence	46 (subject)
Headache	9
With hydrocephalus only	2
With VP shunt in place and hydrocephalus	1
Subject requiring colloid cyst resection	1
Trauma including falls	12
AMS	10
Stroke	3
Assess VP shunt	1
Syncope	1
Vertigo	1
Unknown	9

## Discussion

This study sought to determine the rate at which colloid cysts are missed in brain imaging studies. Over a 10-year period throughout our metropolitan multi-hospital network, we found that 1/5 of colloid cysts had been missed on prior imaging studies. Cysts were missed by both non-neuroradiology-trained imagers (53%) and neuroradiologists (47%) within the network, and on average, it took 3.25 years for the cyst to be diagnosed. We hypothesize that this miss rate is likely an underestimation given that there are likely cases still not detected within our system.

Colloid cysts can be missed on imaging for a variety of reasons, including flow artifacts due to turbulent CSF flow [13]. Diagnosing a colloid cyst paves the way for intervention that is specific and precise to the nature of the colloid cyst. That is, colloid cysts that are more hemorrhagic would need greater care than colloid cysts that are more cystic in nature, which can be aspirated in a minimally invasive manner [8]. Failing to detect the colloid cyst when it has been imaged increases the risk for exploratory surgeries and craniotomies in symptomatic patients. It further diminishes the surgeon’s ability to strategize for an individualized treatment plan.

**Figure 1:**
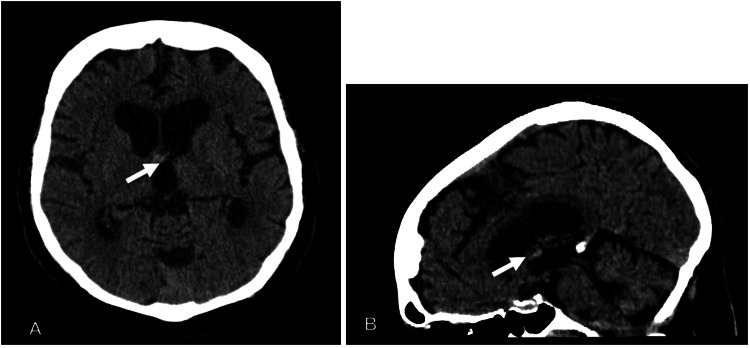
A hyperdense colloid cyst (white arrow) that was missed on the finalized interpretation report (A) CT head axial of study. (B) CT head sagittal of study.

There is great variability in colloid cyst appearance based on composition and size. Some cysts are homogeneous and small and easily lost in the surrounding brain parenchyma, vessels, and CSF at the foramina of Monro. Colloid cyst size ranges from 3 to 40mm; as such, the size of missed colloid cysts is on the lower end of the typical size of colloid cysts [11], which is supported by our study where the average missed lesion measured 5 x 5mm. Their midline location can make detection by the human eye difficult given the lack of asymmetry in the image for the eye to detect. Often, cysts are mistaken for CSF flow artifacts due to turbulent CSF flow in the adjacent lateral and third ventricles on MRI [13]. Additionally, the incidental nature of most colloid cysts decreases the interpreters’ attention to this specific pathology in most cases. Figure 1 demonstrates an example of a colloid cyst depicted in the study but not diagnosed in the interpretive report.

**Figure 2:**
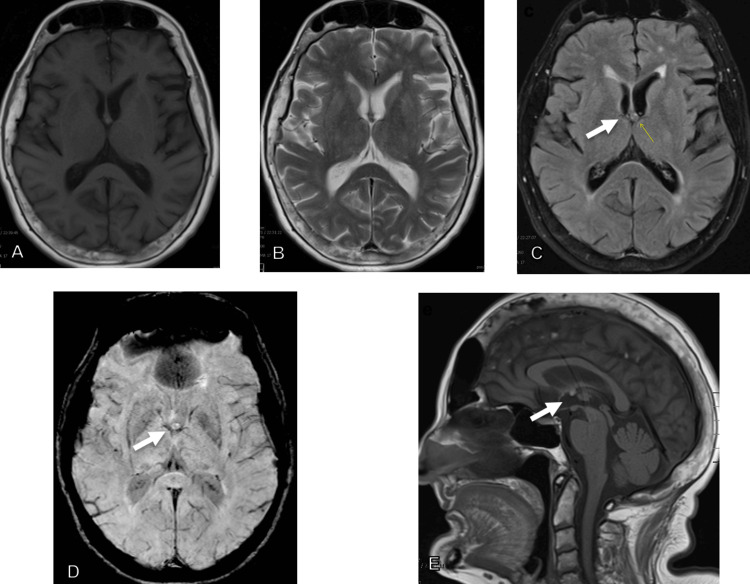
MRI brain study that depicted a colloid cyst (white arrows) that was not reported (A) T1 axial. (B) T2 axial. (C) FLAIR axial. (D) SWI axial demonstrating the black rim susceptibility sign. (E) T1 sagittal.

There are MR brain signs that can be utilized to systematically identify colloid cysts, such as T1-sequence hyperintense focus on MRI at the foramina of Monro. The "black rim susceptibility" sign on the susceptibility weighted imaging (SWI) sequence is a novel finding that can increase radiologists’ detection of colloid cysts in the MRI brain (Figure 2) [12]. Beyond the signs mentioned, future studies evaluating other potential imaging signs and techniques to detect colloid cysts could prove useful. Artificial intelligence software may be helpful to allow automatic detection of these lesions on all head imaging studies based on location, density values, and/or intensity values.

There is clinical significance to missing the diagnosis of a colloid cyst on imaging. Colloid cysts can result in intermittent obstructive hydrocephalus [14], with or without an acute presentation, and have also been implicated in sudden death [15]. In our chart review, we had a case that initially missed the diagnosis of colloid cyst, in which the subject did undergo colloid cyst resection subsequently. This case was followed by a data review in our system. It is likely that there are greater clinical implications to not diagnosing colloid cysts in which a subject received a workup outside of our electronic medical records.

Our study has several limitations. The retrospective nature of the study is an inherent limitation but also a necessity for the type of question we are investigating. Additionally, we do not have pathology in all the cases that we deemed positive for colloid cysts. This is also inherently difficult to obtain, given that many patients with colloid cysts opt to follow their lesions as opposed to resecting on a prophylactic basis. Despite the long time interval and the large hospital network we pulled from, the power of the study would be improved by a study consolidating imaging data from multiple different hospital networks.

## Conclusions

This paper demonstrates the value of increased attention in searching for colloid cysts on both MRI and CT head imaging. The use of concomitant sagittal and coronal imaging on CT, for example, could increase the detection rate of these lesions, noting that cysts often sit in the normally empty space anterior to the massa intermedia on these views.

Simple and small colloid cysts can often be aspirated easily via minimally invasive techniques; however, with time, cysts can enlarge and become more hemorrhagic or internally complex, resulting in the need for more technically difficult resections. Surgical techniques for treatment could include a microsurgical approach, endoscopic fenestration, and stereotactic aspiration, depending on several factors, including the location and content of the lesion. It is imperative to diagnose colloid cysts as early as possible to allow for close imaging follow-up, correlation with symptomatology, and surgical resection as indicated, which is often curative.
